# *Bacillus subtilis* High Cell Density Fermentation Using a Sporulation-Deficient Strain for the Production of Surfactin

**DOI:** 10.1007/s00253-021-11330-x

**Published:** 2021-05-15

**Authors:** Peter Klausmann, Katja Hennemann, Mareen Hoffmann, Chantal Treinen, Moritz Aschern, Lars Lilge, Kambiz Morabbi Heravi, Marius Henkel, Rudolf Hausmann

**Affiliations:** Department of Bioprocess Engineering (150 k), Institute of Food Science and Biotechnology (150), University of Hohenheim, Fruwirthstr. 12, 70599 Stuttgart, Germany

**Keywords:** Surfactin, *Bacillus subtilis*, Biosurfactant, Cyclic lipopeptide, Sporulation

## Abstract

**Abstract:**

*Bacillus subtilis* 3NA is a strain capable of reaching high cell densities. A surfactin producing *sfp*^+^ variant of this strain, named JABs32, was utilized in fed-batch cultivation processes. Both a glucose and an ammonia solution were fed to set a steady growth rate μ of 0.1 h^-1^. In this process, a cell dry weight of up to 88 g L^-1^ was reached after 38 h of cultivation, and surfactin titers of up to 26.5 g L^-1^ were detected in this high cell density fermentation process, achieving a Y_P/X_ value of 0.23 g g^-1^ as well as a q_P/X_ of 0.007 g g^-1^ h^-1^. In sum, a 21-fold increase in surfactin titer was obtained compared with cultivations in shake flasks. In contrast to fed-batch operations using *Bacillus subtilis* JABs24, an *sfp*^*+*^ variant derived from *B. subtilis* 168, JABs32, reached an up to fourfold increase in surfactin titers using the same fed-batch protocol. Additionally, a two-stage feed process was established utilizing strain JABs32. Using an optimized mineral salt medium in this high cell density fermentation approach, after 31 h of cultivation, surfactin titers of 23.7 g L^-1^ were reached with a biomass concentration of 41.3 g L^-1^, thus achieving an enhanced Y_P/X_ value of 0.57 g g^-1^ as well as a q_P/X_ of 0.018 g g^-1^ h^-1^. The mutation of *spo0A* locus and an elongation of AbrB in the strain utilized in combination with a high cell density fed-batch process represents a promising new route for future enhancements on surfactin production.

**Key points:**

• *Utilization of a sporulation deficient strain for fed-batch operations*

• *High cell density process with Bacillus subtilis for lipopeptide production was established*

• *High titer surfactin production capabilities confirm highly promising future platform strain*

## Introduction

Biosurfactants are attracting an increasing interest in both research and industry. The establishment of biosurfactants produced in industrial scale is a clear indication for the transition to environmentally conscious surfactant production. Until now, there is a multitude of different synthetic but bio-based surfactants on the market, many of which are widely used in everyday applications like laundry and dishwashing detergents, dispersants and emulsifiers in food, and cosmetics industries. Compared with biosurfactants, these bio-based solutions often have certain disadvantages, like lower biodegradability, higher toxicity or higher chances of causing skin irritations (Lang and Trowitzsch-Kienast 2002).

Especially microorganisms are used for the production of different biosurfactants. An advantage from the economic point of view is the microbial production of biosurfactants using renewable resources, which ensures the opportunity to avoid the negative environmental impact from conventional surfactants (Henkel et al. 2017). Besides their application as substitutes for conventional surfactants, many microbial biosurfactants furthermore display anti-microbial and anti-fungal properties and are therefore under investigation regarding their application in plant protection (Li et al. 2019). One of the most studied biosurfactant to date is surfactin, a lipopeptide produced by a wide range of *Bacillus* species (Cooper et al. 1981). It consists of a cyclic peptide structured by seven amino acids and a fatty acid moiety of chain lengths between 13 and 15 carbon atoms. The peptide usually possesses l-Glu, l-Leu, d-Leu, l-Val, l-Asp, d-Leu, and l-Leu. An impressive property of surfactin is its capability to reduce the surface tension at water-air interfaces from 72 mN m^-1^ to 27 mN m^-1^ at concentrations as low as 20 mM (Cooper et al. 1981). In *B. subtilis*, surfactin is produced by a non-ribosomal peptide synthase, encoded by the 27 kb comprising *srfA* operon. Expression of this operon is tightly intertwined with the quorum sensing mechanism of *B. subtilis*. Surfactin production is therefore dependent on several physiological states like cell differentiation, growth phase, and cell density (Nakano and Zuber 1991). This complex regulatory mechanism has not yet been completely understood, which is why a strategy for surfactin overproduction relies on decoupling surfactin production from these regulatory circuits by promoter exchanges (Sun et al. 2009; Willenbacher et al. 2016; Wu et al. 2019). Some of these studies have shown that this strategy can yield significant increases in titers. Moreover, further studies described additional effects after increasing the availability of precursor molecules (Liu et al. 2012; Coutte et al. 2015). These insights clarify the complexity of surfactin production and its dependence on different factors.

In consequence of an economic provision of industrially relevant products, a variety of different high cell density fermentation (HCDF) processes were established for several organisms. One major limiting factor in fed-batch operations with *B. subtilis* is its activation of response mechanisms to nutrient limiting conditions. Suboptimal growth conditions result in initiation of different bacterial adaptation strategies (Grossman and Losick 1988; Trach and Hoch 1993; Burkholder and Grossman 2014; Perego and Hoch 2014). Especially sporulation is a highly effective mechanism for *Bacillus* species to survive harsh conditions. However, corresponding endospores are inactive regarding secondary metabolite production (Hoch 1976; Losick et al. 1986). In fed-batch cultivations, growth rate is mostly controlled by nutrient availability. This leads to a limitation of at least one essential nutrient during fed-batch operations, which in turn induces higher sporulation rates, and therefore decreasing amounts of productive cells (Burkholder and Grossman 2014).

To combat limitations of wild-type strains during cultivation in a bioreactor, many strategies of genetic modification have been employed in the past, ranging from targeted modifications (Jung et al. 2012; Wu et al. 2019) to investigation of strains with a reduced genome (Geissler et al. 2019). Especially for application in HCDF processes, Wenzel et al. (2011) established a self-inducible fermentation process using the non-sporulating strain *B. subtilis* 3NA. In this way, a process was designed for heterologous GFP production. The strain 3NA exhibits a frameshift mutation in the *spo0A* gene, leading to a non-sense mutation, as well as a point mutation in the *abrB* locus, elongating the gene product from 96 to 107 amino acids (Reuß et al. 2015). Even though 3NA has a mutation in the *spo0A* gene, it retains its natural competence, which is unusual for *spo0A*-deficient mutants (Losick et al. 1986; Reuß et al. 2015). The reason for this is most likely due to the second mutation of AbrB. Spo0A is an important transcriptional regulator and controls several genes involved in sporulation initiation (Green et al. 1991; Bird et al. 1993). Accordingly, mutation of *spo0A* results in strains incapable of sporulation (Hoch 1976; Losick et al. 1986). Simultaneously, Spo0A acts as transcriptional repressor of the *abrB* gene (Strauch et al. 1990)*.* AbrB is a transcriptional regulator for several genes, which are important during transition state between exponential and stationary phase (Strauch et al. 1989). In this context, AbrB acts as a transcriptional repressor for *srfA* operon. Hence, inactivation of *spo0A* leads to overexpression of *abrB*, which in turn leads to repression of the surfactin operon expression.

Wang et al. (2020) reported on *B. subtilis* TS1726 strain, which encodes a strong Pg3 promoter for improved surfactin production. To verify the influence of sporulation on surfactin production, different non-sporulating derivatives (TS1726 Δ*spo0A*, Δ*spoIIIE* or Δ*spoIVB*) were analyzed. Interestingly, only Δ*spo0A* deletion mutant produced absolutely no surfactin. Furthermore Wang et al. (2020) described that deletion of *spoIVB* in combination with the overexpression of the genes *leuABCD* and *ilvK* resulted in a surfactin titer of 11.3 g/L in shake flask cultures on a complex medium containing 1 g L^-1^ yeast extract. By adding 5 g L^-1^ leucine to the culture medium, they achieved a surfactin titer of 16.7 g L^-1^ after 48 h.

In this work, a fed-batch process is reported using JABs32, an *sfp*^*+*^ version of the 3NA strain, for surfactin production in a mineral salt medium with glucose as a carbon source. Outcomes will be compared and discussed with the well-established *sfp*^+^ version of 168 strain, named JABs24.

## Materials and methods

### Bacterial strains

The strains used in this study were *B. subtilis* strains 168 and 3NA (Wenzel et al. 2011). In both strains, a mutation within the *sfp* gene impeded surfactin production (Reuß et al. 2015). Therefore, an *sfp*^*+*^ *trpC ΔmanPA* variant was created for both 168 and 3NA, labeled JABs24 and JABs32, respectively (Geissler et al. 2019). The bacterial strains were kindly provided by Dr. Josef Altenbuchner, Institute for Industrial Genetics, University of Stuttgart, Germany. *B. subtilis* 3NA is available from the Bacillus Genetic Stock Center (BGSCID 1S1).

### Media and conditions for cultivation

The first precultures were performed in LB-medium with 10 g L^-1^ tryptone, 5 g L^-1^ yeast extract, and 5 g L^-1^ NaCl. The second preculture was inoculated in the respective cultivation medium of the main culture. Media used for fermentation processes were described by Willenbacher et al. (2015) and Wenzel et al. (2011). For a two-step fermentation process, a variation of the mineral salt medium (MSM) from Willenbacher et al. (2015) was used, consisting of 5.5 g L^-1^ glucose x H_2_O, 4 g L^-1^ Na_2_HPO_4_, 14.6 g L^-1^ KH_2_PO_4_, 4.5 g L^-1^ (NH_4_)_2_SO_4_, 0.2 g L^-1^ MgSO_4_ x 7 H_2_O, and 3 mL L^-1^ trace element solution (TES). TES contained 40-mM Na_3_citrate, 5-mM CaCl_2_, 50-mM FeSO_4_, and 0.6-mM MnSO_4_ x H_2_O. The pH of the media used for shake flask cultivations was adjusted to 7.0.

All shake flask experiments were carried out as batch cultivations in an incubator shaker (NewbrunswickTM/Innova 44, Eppendorf AG, Hamburg, Germany) set to 37 °C and 120 rpm. All bioreactor cultivations were performed in a 30-L fermenter (ZETA GmbH, Graz/Lieboch, Austria) filled with 12-L batch medium. The temperature was set to 37 °C, pH to 7.0, and initial stirrer speed to 300 rpm using three Rushton turbines. Dissolved oxygen was set to a minimum of 50% by adjusting stirrer speed and aeration rate. Initial aeration rate during batch phase was set to 2 L min^-1^ and adjusted to 10 L min^-1^ when feeding 50% (w/w) glucose solution. All experiment data shown was derived from experiments performed in duplicates.

### Shake flask cultivation

For Preculture I, 25 mL of LB medium were inoculated and incubated for 13 h in a 100-mL baffled shake flask. Preculture I was used to inoculate Preculture II to an OD_600_ of 0.1 in MSM or high cell density medium (HCDM) from Wenzel et al. (2011) with 25 g L^-1^ glucose as the sole carbon source. After 8 h incubation at 37 °C, the bacterial suspension was transferred into 100 mL of the respective medium in a 1-L baffled shake flask to an OD_600_ of 0.1. Subsequently, samples were taken every 3 h during cultivation process.

### Bioreactor fermentation

A glycerol stock was used to inoculate 25 mL LB medium with the strains JABs24 or JABs32, respectively. Preculture I was inoculated for 13 h at 37 °C and 120 rpm. Next, the bacterial suspension was used to inoculate 200 mL HCDM in Preculture II to an OD_600_ of 0.1, which was then cultivated for 8 h at 37 °C and 120 rpm when JABs32 was used or for 12 h at 37 °C and 120 rpm when JABs24 was used, due to its reduced cell growth.

**Fig. 1:**
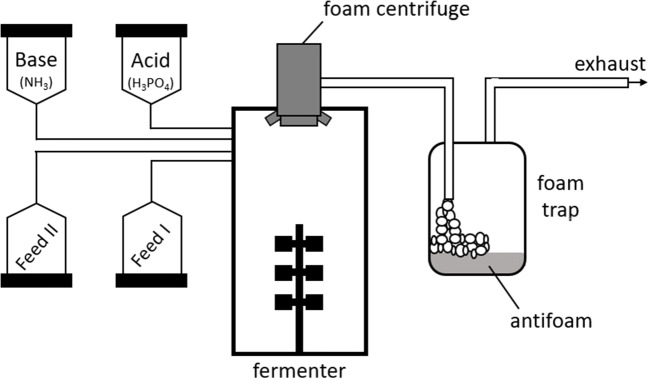
Illustration of the bioreactor set-up. Shown is the fermenter in the middle with a foam centrifuge at the exhaust port. The exhaust pipe was bypassed through canister used as a foam trap with antifoam agent Contraspum A4050 in 3 L of water. The air could freely leave the canister through a second exhaust port leading to the exhaust filter. Four scales were used to monitor pH control as well as feeding solutions. In case of a two-feed process, one of the feeds was swapped after the first fed-batch phase for the second glucose feed

A 30-L bioreactor was used in this study. The initial volume of the reactor was 12 L of HCDM or two-step batch medium, respectively. The control of pH was ensured by using 4 M H_3_PO_4_ and 20 % (v/v) NH_3_ solutions. A foam centrifuge was employed as a means of mechanic foam disruption at a speed of 2960 rpm. As a second means of overfoaming protection, antifoam agent Contraspum A4050 (Zschimmer & Schwarz GmbH, Lahnstein, Germany) was used, controlled by a foam sensor in the exhaust gas pipe. To catch potentially overfoaming and surfactin-enriched solutions, a foam trap was installed in front of the exhaust gas filter. The foam trap was a container with a capacity of 25 L, filled with 3 L of water containing 20 mL antifoam agent Contraspum A4050. An illustration of the bioreactor set-up is shown in Fig. 1.

For the HCDM-based approach, Preculture II was used to inoculate a volume of 12 L to an OD_600_ of 0.1. The strains were grown overnight at 37 °C, pH 7, and an aeration rate of 2 L min^-1^. Afterwards, samples were taken every 2 h after cultivation time of 12 h. When glucose was depleted, the fed-batch phase was started by introducing two feed solutions. Feed I consisted of 50% (w/w) glucose, 12 g L^-1^ MgSO_4_, and 120 mL L^-1^ TES, while Feed II was comprised of 396 g L^-1^ (NH_4_)_2_HPO_4_. Altogether, 6 L of Feed I and 2 L of Feed II were used. An exponential feed was utilized at a steady growth rate of 0.1 h^-1^. The feed rates for Feed I and II were calculated using the following equation:

1 $$ F(t)=\left[\left(\frac{\mu }{Y_{X/S}}\right)+m\right]\ast \left[\frac{c_{x_0}\ast {V}_0}{c_{s_0}}\right]\ast {e}^{\mu } $$

In this equation F(t) is the feed rate (kg h^-1^); μ the growth rate (h^-1^), set to 0.1 h^-1^; m the maintenance coefficient 0.04 g g^-1^ h^-1^; ${c}_{x_0}$ the biomass concentration at feed start (g L^-1^); V_0_ the bioreactor volume in L; and ${c}_{s_0}$ the glucose concentration in Feed I (g L^-1^). The initial feed rate of Feed II was set to 20% of that of the initial rate of Feed I. The fed-batch process was run until Feed I was depleted. Samples were taken every 2 h.

The two-step process used a batch medium based on MSM, but with 5 g L^-1^ glucose as carbon source. A 2-L solution with 150 g L^-1^ glucose was fed after the initial glucose was consumed. Afterwards, a 5 kg 50 % (w/w) glucose solution with 60 mL L^-1^ TES and supplemented with 12 g L^-1^ MgSO_4_ was used for the second feeding phase. As nitrogen source, a 1.5 L comprising 396 g L^-1^ (NH_4_)_2_HPO_4_ solution was prepared and was fed into the bioreactor at a rate of 30 % of that of the glucose feed.

### Analysis

Before analysis, samples were centrifuged for 10 min at 3890 g for biomass removal. Glucose was quantified using enzymatic assay kits (R-Biopharm AG, Darmstadt Germany, Cat. No. 10148261035). Ammonia concentration was measured using a photometric ammonia test kit (Merck KGaA, Darmstadt, Germany, Cat. No. 1.14752.0001). CDW was calculated by multiplying the OD_600_ values with a factor previously determined by drying the biomass for 48 h at 110 °C and weighing the dried biomass. For JABs24 the factor was determined at 0.322 and for JABs32 the factor was 0.372.

### Surfactin quantification

Surfactin was quantified by HPTLC analysis (CAMAG AG, Muttenz, Switzerland), using the previously reported procedure by Geissler et al. (2017). A volume of 2 mL cell-free supernatant was extracted three times with chloroform/methanol (2:1). After each extraction, the solvent layers were pooled and dried afterwards using a rotary evaporator at 10 mbar and 40 °C. The dried samples were resolved in 2-mL methanol and applied in 6 mm bands on a silica HPTLC plate. A surfactin standard obtained from Sigma Aldrich was applied in a range from 30 to 600 ng. As a mobile phase, chloroform/methanol/water (65:25:4) was used with a migration distance over 60 mm. The plate was analyzed at 195 nm for surfactin detection.

### Data analysis

The yield of biomass per substrate (Y_X/S_), product per biomass (Y_P/X_), growth rate μ, and specific productivity (q_P/X_) were determined using the equations shown below. Plotted were the glucose and ammonia concentrations, as well as CDW and surfactin titers for every sampling time point. Y_X/S_ was determined at the maximum CDW, while Y_P/S_ and Y_P/X_ were determined at the maximum surfactin concentrations. Specific productivity q_P/X_ was split into two calculations. Equation 4 describes the productivity of biomass of the whole process, taking maximum CDW and surfactin concentrations into consideration. Equation 5 on the other hand describes the productivity over time, taking values from the fitting curve.

2 $$ {Y}_{X/S}={\left.\frac{X}{S}\right|}_{X= Xmax} $$

3 $$ {Y}_{P/S}={\left.\frac{P}{\Delta S}\right|}_{P= Pmax} $$

4 $$ {q}_{P/X,\kern0.5em overall}=\frac{P_{max}}{X_{P_{max}}\cdotp \Delta t} $$

5 $$ {q}_{P/X}(t)=\frac{\Delta P}{X\cdotp \Delta t} $$

6 $$ {Y}_{P/X}={\left.\frac{P}{X}\right|}_{P= Pmax} $$

7 $$ \mu =\frac{\ln \left({x}_2\right)-\ln \left({x}_1\right)}{\Delta  t} $$

The fitting curves shown in all figures were derived using scientific graphing and data analysis software (SigmaPlot, Systat Software Inc., San Jose, CA). A logistic equation with four parameters was used to fit the data for the bacterial growth under limiting conditions (Zwietering et al. 1990). A logistic model for biomass growth was shown to be suitable for the description of biomass during biosurfactant producing processes (Sudhakar Babu et al. 1996; Ramana et al. 2007; Henkel et al. 2014).

They were generated using the dynamic fit function of SigmaPlot14, choosing a 4-parameter logistic fit. Growth rate μ and q_P/X_ were then calculated using the generated fit values.

## Results

### Batch cultivation in shake flasks

**Fig. 2:**
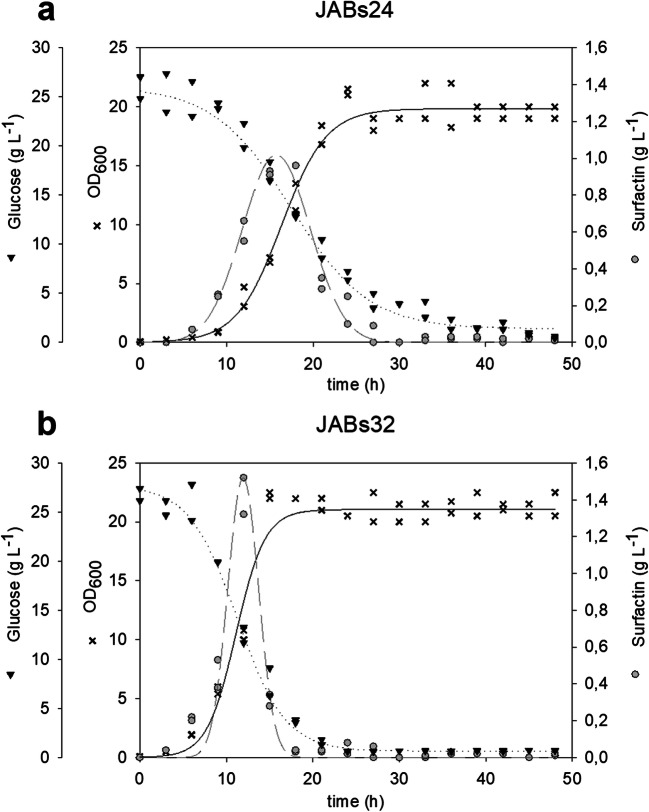
Batch cultivations in shake flasks with strains JABs24 (**a**) and JABs32 (**b**) in MSM with an initial glucose concentration of 25 g L^-1^. Plotted are optical density (black crosses), surfactin concentrations (grey circles), and glucose (black triangles) over time

Shake flask cultivations of JABs24 and JABs32 were conducted in MSM (Fig. 2a and Fig. 2b) as well as in HCDM. In both cultivations, 25 g L^-1^ glucose were used as sole carbon source. Comparison of bacterial growth in batch cultivations using MSM for JABs24 and JABs32 showed a significantly faster growth of the sporulation-deficient JABs32 strain. Average growth rates of 0.36 h^-1^ and 0.22 h^-1^ for JABs32 and JABs24, respectively, have been recorded. A maximum OD_600_ of 22.5 was reached for JABs32 after 15 h of cultivation. In contrast, JABs24 reached its maximum OD_600_ of 21.5 after 24 h of cultivation.

A comparison of produced surfactin concentrations revealed that JABs24 reached its maximum during early exponential phase, and declining values were detected during late exponential and stationary phase (Fig. 2a). Similarly, JABs32 reached its maximum surfactin concentration at similar time points of 15–18 h, after which the concentration fell rapidly to concentrations below 50 mg L^-1^ after 24 h. Peak surfactin concentrations were also observed during exponential phase in JABs32 (Fig. 2b). With respect to surfactin concentrations produced by *B. subtilis* strains JABs32 and JABs24, the non-sporulating JABs32 reached 21 % higher values compared with JABs24 (1.47 g L^-1^ versus 1.21 g L^-1^,).

Comparison of HCDM-based cultivation with MSM yielded lower OD_600_ values for both strains, as well as lower surfactin concentrations. JABs32 produced a maximum of 1.12 g L^-1^ of surfactin, while JABs24 produced 0.94 g L^-1^ with OD_600_ values of 19.7 and 18.8, respectively. Glucose was the growth-limiting factor in MSM, as ammonium was detectable until the end of cultivation after 48 h. In contrast, ammonium was growth limiting in HCDM.

### Fed-batch fermentation

**Fig. 3:**
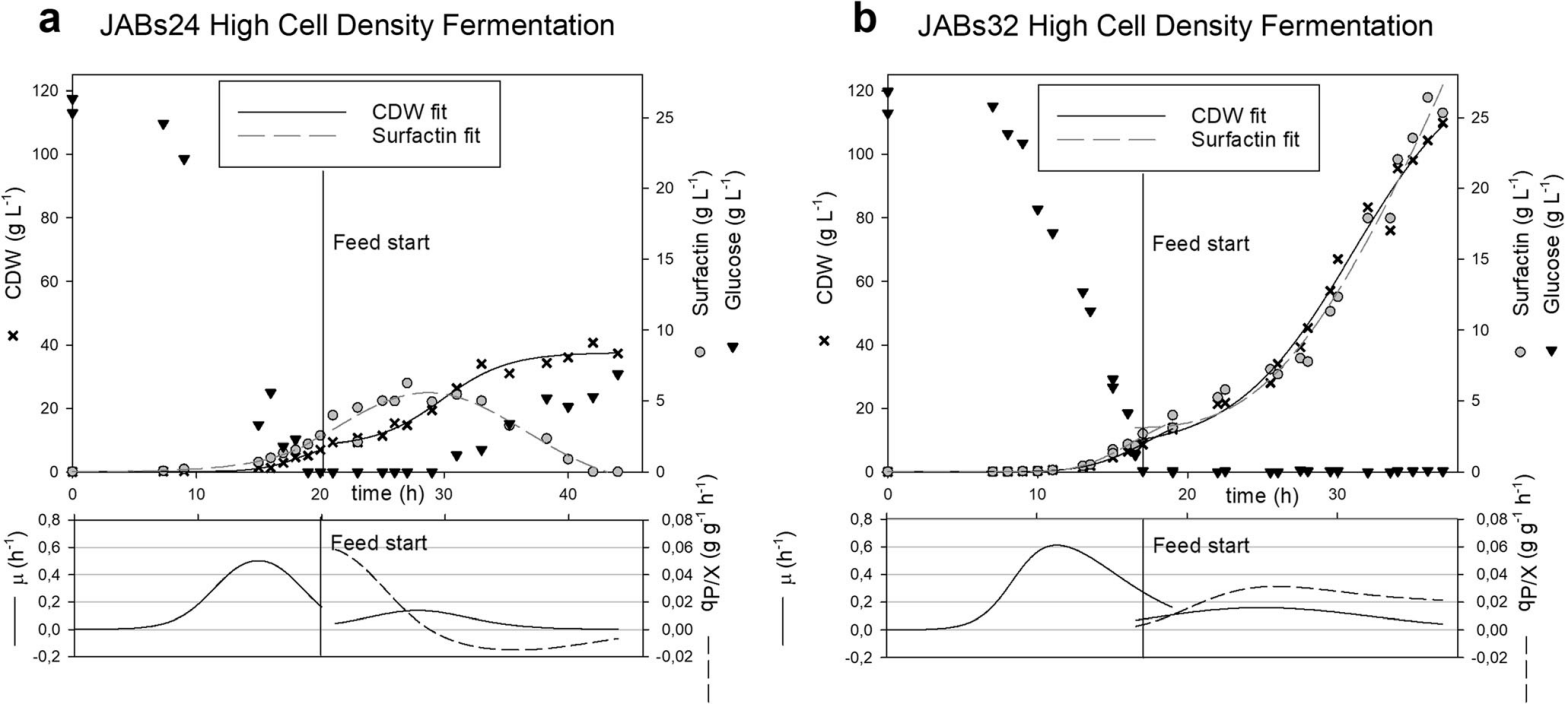
Fed-batch bioreactor fermentations with strains B. subtilis JABs24 (**a**) and JABs32 (**b**) in HCDM with an initial glucose concentration of 25 g L^-1^. Plotted on the top graphs are optical density (black crosses) and surfactin concentrations (grey circles) over time. The bottom graphs display growth rate μ (black line) and specific productivity q_P/X_ (broken line) over time

In this approach, a fed-batch process was conducted to investigate the production of surfactin by JABs32 in HCDM. Using a glucose and an (NH_4_)_2_HPO_4_ feed, OD_600_ values of up to 260 could be reached after 38 h of cultivation using strain JABs32, which corresponds to a CDW of 88 g L^-1^ (Fig. 3b). In comparison, JABs24 only reached an OD_600_ of up to 112 after 45 h, corresponding to a CDW of approx. 38 g L^-1^ under the same conditions (Fig. 3a). After JABs24 growth decreased, glucose accumulation was observed in the fermentation broth, and a maximum surfactin concentration of 6.25 g L^-1^ was detected. This concentration started decreasing after 39 h when stationary growth phase was reached and was reduced to 0.96 g L^-1^ at the end of the fermentation process (Fig. 3a). In contrast, JABs32 produced a maximum of 26.9 g L^-1^ surfactin using this process, and surfactin production increased steadily until the glucose feed was depleted (Fig. 3b).

**Fig. 4:**
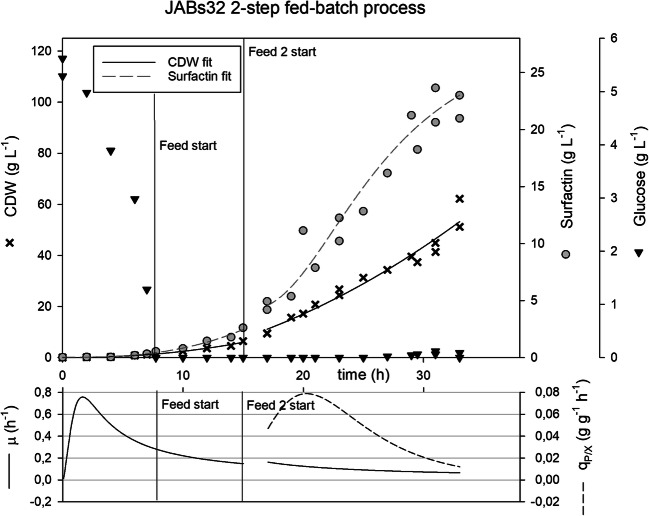
Two-step fed-batch bioreactor fermentations with strain JABs32 in optimized MSM with a glucose and ammonium phosphate feed. Plotted on the top graph are optical density (black crosses) and surfactin concentrations (grey circles) over time. The bottom graph displays growth rate μ (black line) and specific productivity q_P/X_ (broken line) over time

Comparison of calculated values of specific yields of both strains showed that at the beginning of fed-batch phase JABs32 reached Y_P/X_ values of about 0.07 to 0.09 g g^-1^ (Eq. 6) which remained stable until the end of the fermentation process (Fig. 4). JABs24 on the other hand exhibited a steady decrease until surfactin was finally degraded. After the beginning of the fed-batch phase, calculated maximum growth rates (Eq. 7) of JABs24 and JABs32 were 0.1 h^-1^ and 0.17 h^-1^ and were reached after about 3 h and 5 h, respectively. The most important process parameters are also summarized in Table 1.

**Table 1:** Overview of different surfactin production processes described in literature compared to JABs24 as a reference strain and JABs32 in both cultivation processes. Strains and processes were compared with regard to maximum product concentration P_max_, product yield per biomass Y_P/X_, product yield per glucose Y_P/S_, overall and maximum growth factor μ and μ_max_, overall and maximum specific productivity q_P/X_, and q_P/X, max_. Data shown were taken from this study, as well as from publications by Willenbacher et al. (2014), Jiao et al. (2017), Hu et al. (2020), Geissler et al. (2019), Wang et al. (2019), Wu et al. (2019), Coutte et al. (2013), and a patent filed by Kaneka Corp., Minako, Japan (Yoneda et al. 2006)

Process, strains	P_max_	Y_P/X_	Y_P/S_	μ	μ_max_	q_P/X_max	q_P/X,overall_	Reference
carbon source	(g ∙ L^-1^)	(g ∙ g^-1^)	(g ∙ g^-1^)	(h^-1^)	(h^-1^)	(g ∙ g^-1^ ∙ h^-1^)	(g ∙ g^-1^ ∙ h^-1^)	
*168 (JABs24) batch on glucose*	*2.56*	*0.35*	*0.07*	*0.29*	*0.49*	*0.36*	*0.018*	*This study*
*3NA (JABs32) batch on glucose*	*2.68*	*0.26*	*0.08*	*0.39*	*0.61*	*0.19*	*0.018*	*This study*
*3NA (JABs32) 2-step feed on glucose*	*2.61*	*0.41*	*0.12*	*0.4*	*0.75*	*0.14*	*0.027*	*This study*
*168 (JABs24) fed-batch on glucose*	*6.25*	*0.17*	*0.038*	*0.07*	*0.14*	*0.05*	*0.007*	*This study*
*3NA (JABs32) fed-batch on glucose*	*26.4*	*0.23*	*0.16*	*0.12*	*0.16*	*0.03*	*0.007*	*This study*
*3NA (JABs32) 2-step feed 2 on glucose*	*23.7*	*0.57*	*0.16*	*0.12*	*0.16*	*0.07*	*0.018*	*This study*
*DSM 1090 batch fermentation process* *with foam fractionation on glucose*	*0.9*	*0.22*	*0.18*	*n.a.*	*n.a.*	*n.a*	*0.008*	Willenbacher et al. 2014
*THY-7 batch fermentation process with foam recovery on sucrose*	*9.74*	0.92*	0.14*	0.15*	*n.a.*	*n.a.*	0.025*	Jiao et al. 2017
*168 batch nonbuffered cultivation* *on corncob hydrolysate (mainly xylose)*	*2.074*	0.93*	0.17*	0.18*	*n.a.*	*n.a.*	0.04*	Hu et al. 2020
*168 (JABs24) anaerobic fermentation on* *glucose*	*0.296*	0.95***	*0.18*	0.02*	*n.a.*	*n.a.*	0.012***	Geissler et al. 2019
*168 cultivation with systematic gene repression on sucrose*	*0.75*	*0.31*	*n.a.*	*0.18*	*n.a.*	*n.a.*	*0.013*	Wang et al. 2019
*168 systematic genetic engineering* *on sucrose*	*9.2*	1*	*n.a.*	0.12*	0.3*	0.23*	0.02*	Wu et al. 2019
*168 continuous bioproduction* *Process on glucose*	7.1**	0.55*	*n.a.*	*n.a.*	*n.a.*	*n.a.*	0.011*	Coutte et al. 2013
*Kaneka Corp. (Japan) patent on soybean flour*	*50*	*n.a.*	*n.a.*	*n.a.*	*n.a.*	*n.a.*	*n.a.*	Yoneda et al. 2006, *patent no. US7011969B2*

*values were calculated using the presented data in their respective publications

**value represents absolute surfactin concentration as opposed to concentration in grams per volume

***Y_P/X_ and q_P/X_ were calculated differently in this work and therefore converted to the format of this publications

### Two-stage feed process

After evaluation of different media in batch cultivations, a two-step feeding process was developed on the basis of MSM. One reason for this approach was that control of growth at an early stage of fermentation reduces oxygen consumption and therefore stirrer speed, which reduces foam formation and use of antifoam agents. After initial glucose was consumed, a 2-L glucose feed with a concentration of 150 g L^-1^ was introduced to reach a CDW high enough to start the main fed-batch process. After this glucose feed was depleted, second glucose and (NH_4_)_2_HPO_4_ feeds were introduced as described in the previous process. Only JABs32 was chosen for this process as it was a promising strain for high cell density fermentation and demonstrated more promising surfactin production capabilities.

Figure 4 shows the time course of the two-step fermentation process for JABs32. In this fermentation, JABs32 reached OD_600_ values of up to 182 or CDW of 60 g L^-1^ after 32 h. Surfactin concentrations reached values of up to 23.7 g L^-1^. Both, CDW and surfactin concentration exhibited a nearly linear increase after start of feed 2. After the initial 5 g L^-1^ glucose was depleted, no glucose accumulation could be observed during the fermentation process. The glucose feed II and ammonia feed bottles were depleted after a cultivation time of 32 h, and the process was terminated by this.

The bottom part of Fig. 4 shows the growth rate during batch and fed-batch, as well as the specific productivity q_P/X_ for the fed-batch phase over time. From the beginning of feed 2 until 25 h, a gradual decrease of growth rate (Eq. 7) from 0.2 h^-1^ to 0.08 h^-1^ was derived. The q_P/X_ (Eq. 5) decreased during the fermentation process from its maximum at 20 h of 0.08 to 0.01 g g^-1^ h^-1^ at the end of the process. Overall q_P/X_ (Eq. 4) was at 0.07 g g^-1^ h^-1^ in this fermentation process, as summarized with other important process parameters in Table 1.

## Discussion

Batch cultivations showed that sporulation-deficient *B. subtilis* strain JABs32 is a promising bacterial system for increased surfactin production compared with the respective sporulating JABs24 strain. In both mineral salt media, MSM and HCDM, JABs32 was able to produce 21 % higher surfactin concentrations in batch fermentations than JABs24. Moreover, JABs32 exhibited faster cell growth (0.1 h^-1^ vs. 0.17 h^-1^) and reduced lag phases. The sporulation deficiency might be one reason for the higher surfactin productivity compared with JABs24. Spores are unproductive hibernation-like cells and are usually the result of adverse conditions or nutrient limitation, as well as a product of cell density dependent differentiation control (Grossman and Losick 1988; Trach and Hoch 1993). Although no spore formation could be observed microscopically in any case, an initiation of cell differentiation in the strain JABs24 and a related beginning of sporulation would be a possible explanation for this observation.

In both strains, surfactin was rapidly degraded when nutrients were limited. This could be due to consumption of surfactin as a nutrient source. As a lipopeptide, the fatty acid moiety as well as the amino acids in the cyclic peptide could be metabolized during nutrient limiting conditions, as they are potentially suitable carbon and nitrogen sources for *Bacillus subtilis*.

Bioreactor cultivations confirmed the superior surfactin production capabilities of JABs32 compared with JABs24. Surfactin titers were up to 4.2-fold higher at the end of fed-batch fermentation in the non-sporulating strain, while cell density was doubled (Fig. 3a and Fig. 2b) compared with the highest surfactin concentration in JABs24.We hypothesize that when cell density reaches a threshold range, cell differentiation facilitated by a functional *abrB*-gene such as spore formation resulting in growth reduction and entering of stationary phase is the preferred strategy (Losick et al. 1986; Grossman and Losick 1988; Shank and Kolter 2011). Presumably, cell differentiation reduced the number of surfactin-producing cells, resulting in lower productivity of the entire population. As observed in batch fermentations, total surfactin concentrations decreased after reaching a maximum during exponential phase for both strains JABs24 and JABs32. This decline was also detectable in JABs24 fed-batch processes even though glucose and ammonia were beginning to accumulate, which in turn indicates that the carbon and nitrogen sources were not a limiting factor. This implies that surfactin is not degraded due to a lack of nutrient sources and metabolization of its constituents, but due to a shift of genetic expression patterns during stationary phase or surfactin is degraded throughout the entire fermentation process but only during stationary phase degradation is faster than production.

In contrast to JABs24, up until biomass concentrations of 88 g L^-1^ JABs32 only reached a stationary phase when glucose was depleted. It is not clear at which point this strain experiences limitation-based effects on its growth behavior due to extreme biomass concentrations, but it is likely to be limited by the viscosity of the medium at high CDW and ensuing limitations in nutrient availability. At concentrations observed in this study, viscosity of the medium was already observed to be high, which led to difficulties in downstream processing, where biomass was separated by centrifugation. Therefore, a process is more feasible with lower biomass concentrations at the end of cultivation. The two-feed strategy with MSM medium optimized for high cell density cultivations achieved similar surfactin concentrations in a shorter time frame compared with the single feed strategy (Fig. 4). In this way, about 40% less biomass concentrations were measured after 29 h resulting in positively affected yields and specific productivities. Many processes use the foaming capabilities of surfactin producing cultures for product enrichment (Davis et al. 2001; Willenbacher et al. 2014; Jiao et al. 2017). This leads to loss of culture medium and productive cells due to overfoaming of the reactor. It also leads to challenging feeding solutions during fed-batch operations, due to shifting reactor volumes and cell densities (Chenikher et al. 2010). This process employed foam centrifuges and antifoam solutions to tackle these issues. Nevertheless, antifoam agents are often considered expensive and impede downstream processing. Future works should focus on incorporating foam fractionation techniques in high cell density fermentations to tackle this issue. Batch cultivations with differing concentrations of antifoam agent have shown that the concentrations introduced during fed-batch processes do not affect growth and surfactin production.

The inactive variant of *spo0A* disrupts spore formation in JABs32 (Losick et al. 1986; Green et al. 1991; Wenzel et al. 2011). Usually this also leads to drastically decreased natural competence and surfactin titers as reported by Wang et al. (2020) for *B. subtilis* TS1726 Δ*spo0A* which displayed no surfactin formation. Accordingly, this master regulator for sporulation initiation seems to be essential for surfactin production in *B. subtilis*. In contrast, the strain JABs32 exhibits a nonsense mutation in the *spo0A* gene and produces notable surfactin amounts suggesting that *abrB* elongation could have a compensatory effect. In *B. subtilis* wild type, and accordingly in JABs24 the expression of AbrB inhibits surfactin production, while Spo0A inhibits AbrB expression (Strauch et al. 1989; Strauch et al. 1990). This leads to the hypothesis that the elongated *abrB* genotype leads to an inactive AbrB variant, as no inhibition of surfactin production could be observed in JABs32, as would be the case in a ∆*spo0A* mutant with active AbrB.

Comparison of growth rate μ and specific productivity q_P/X_ implies a correlation between these parameters. During batch phase, where growth rates were at their peaks, surfactin productivity also reached its highest value as seen in Fig. 3b and Fig. 4. The same circumstances could be observed during the fed-batch process, where productivity and bacterial growth decreased over time. In JABs24, this phenomenon even leads to decline of surfactin when growth rate started decreasing. At the same time this could be attributed to lower oxygen saturation in the fermentation broth, due to higher OUR at high cell densities. Studies already showed that oxygen supply played a crucial role in surfactin production (Coutte et al. 2010; Willenbacher et al. 2015; Hoffmann et al. 2020).

Comparison of Y_P/X_ values of JABs24 and JABs32 indicates that sporulation negatively effects surfactin productivity during fed-batch processes, which was also shown by Wang et al. (2020).

When stationary phase approached, surfactin produced by JABs24 started to decrease and therefore reduced its yield coefficient. JABs32 however maintained a stable yield coefficient during fed-batch phase in both processes. The two-step process had on average a 30 % increase in yield per biomass when compared with the single feed process. Additionally, growth rates were closer to the desired growth rate of 0.1 h^-1^ when the two-step system was employed. It is not yet clear as to why JABs32 maintains a higher growth rate at the same feeding profile in different media. One reason could be that the difference in media composition leads to a more efficient Y_X/S_ and therefore less glucose is needed to maintain higher growth rates.

Table 1 shows a comparison of the established high cell density fermentation process compared with other surfactin producing processes in literature.

The non-sporulating JABs32 strain exhibits the highest titer (26.9 g L^-1^) of surfactin in this short of a timespan (38 h). As can be extrapolated from the development of the biomass and surfactin titer in Fig. 3b, even higher titers can presumably be achieved. The high achieved concentrations also clearly show that no significant product inhibition is observed in the covered concentration range. However, this must be quantified more precisely in further studies.

The patent filed by Kaneka Corp. describes a process using soybean flour as a carbon source and reaching surfactin concentrations of up to 50 g L^-1^ after 80 h; however, after 32 h this process describes surfactin concentrations of 18 g L^-1^ (Yoneda et al. 2006). This patent does not sufficiently describe the essential parameters of process control and methods of quantification of the product. Furthermore, neither efficiency parameters nor yields are mentioned therein. After 32 h, JABs32 reached concentrations of up to 23.7 g L^-1^ using a two-step fermentation process. Additionally, this process was carried out in a defined mineral salt medium without supplementation of amino acids or yeast extract compared with this patent. Comparison of this process with new established batch fermentations using genetically modified strains also shows that in a shorter timespan higher surfactin concentrations could be reached. This becomes especially obvious when comparing Jiao et al. (2017) and Wu et al. (2019) to the two-step process, where both strains exhibited about 50% longer fermentation times (Table 1). Establishing some of these described mutations in a non-sporulating strain could further improve the currently achieved results.

Comparing the overall productivity q_P/X_ of different fermentation processes for surfactin production, the implemented processes are comparable at the end of batch phases with batch processes described in literature. The genetic engineering process described by Wu et al. (2019) resulted in mutant strains that were reported to be capable of producing more surfactin than their reference strain shown in the table. However, no data on growth and CDW were available for these strains. A continuous process described by Coutte et al. (2013) led to the successful implementation of a microfiltration process coupled to a bubbleless membrane bioreactor. In this way, 7.1 g of surfactin were produced using a 3 L bioreactor after 48 h of cultivation. This resulted in a q_P/X_ of 0.11 g g^-1^ h^-1^. Compared to all those processes, JABs32 fed-batch fermentation has the advantages of using a defined mineral salt medium with little costs, producing highest titers of surfactin in a short timeframe without additional expensive hardware. Both processes with the sporulation-deficient strain exhibit high surfactin titers in fed-batch operations, and the production trend indicates that even higher titers are conceivable in extended processes.

This work has shown that high cell density fermentation processes are promising tools for enhanced surfactin production without the need of addition of either peptone, tryptone, or pure leucine. One way of achieving the high biomass concentrations described in this work is the utilization of sporulation deficient mutants of *B. subtilis*. Due to the absence of *spo0A* and elongation of *abrB*, the strain JABs32 demonstrated much higher surfactin production capabilities in fed-batch cultivation compared with the laboratory strain 168 (JABs24). With the potential to be an efficient production strain in high-cell density processes as well as its unique suitability to serve as an accessible host for genetic modifications, JABs32 is a promising candidate for both future process as well as strain development.

## Data Availability

All discussed data have been included into the manuscript. Please turn to the corresponding author for all other requests.
